# Unveiling the Intricate Causal Nexus Between 91 Circulating Inflammatory Proteins and Perianal Abscess Through a Comprehensive Bidirectional Two‐Sample Mendelian Randomization Analysis

**DOI:** 10.1002/hsr2.70803

**Published:** 2025-05-29

**Authors:** Zehui Wang, Tian Chen, Hongshuo Shi, Xuecheng Zhang, Wei Yang

**Affiliations:** ^1^ Shuguang Hospital Affiliated to Shanghai University of Traditional Chinese Medicine Shanghai China; ^2^ China‐Japan Friendship Hospital Beijing China

**Keywords:** 91 circulating inflammatory proteins, causal effect, genetic association, Mendelian randomization, perianal abscess

## Abstract

**Background:**

The link between circulating inflammatory proteins and perianal abscess remains uncertain. This study utilized the Mendelian randomization approach to examine the potential causality of 91 circulating inflammatory proteins in relation to perianal abscess (PA) and assessed the bidirectionality of these causal effects.

**Methods:**

We conducted analyses using Genome‐wide Association Studies (GWAS) summary statistics for 91 circulating inflammatory proteins, sourced from 11 cohorts with a combined total of 14,824 participants. Genetic associations for PA were taken from the FinnGen Consortium R9 data, consisting of 2595 PA cases and 301,931 control individuals. Two‐sample Mendelian randomization (MR) analysis was utilized to investigate the causal links between these inflammatory proteins and PA. To validate the reliability of our MR findings, sensitivity analyses were carried out. Furthermore, reverse Mendelian randomization was employed to assess whether there is evidence for a reciprocal causal effect.

**Results:**

Employing the inverse‐variance weighted (IVW) approach, our findings identified 7 circulating inflammatory proteins with a potential causal link to PA. Notably, increased levels of interleukin‐18 receptor 1 (OR = 1.10, 95% CI 1.01–1.19, *p* = 0.039), interleukin‐33 (OR = 1.31, 95% CI 1.10–1.56, *p* = 0.002), interleukin‐7 (OR = 1.32, 95% CI 1.06–1.64, *p* = 0.014), and tumor necrosis factor ligand superfamily member 12 (OR = 1.16, 95% CI 1.01–1.32, *p* = 0.032) showed associations with increased risk for PA. Conversely, higher levels of T‐cell surface glycoprotein CD6 isoform (OR = 0.81, 95% CI 0.68–0.98, *p* = 0.026), interleukin‐2 (OR = 0.80, 95% CI 0.66–0.97, *p* = 0.026), and programmed cell death 1 ligand 1 (OR = 0.84, 95% CI 0.70–1.00, *p* = 0.047) appeared to have protective effects against this condition. Additionally, our analysis has not found any evidence to suggest a reverse causal relationship between PA and the 7 specific inflammatory factors studied.

**Conclusions:**

Our Mendelian randomization analysis identifies a probable causal association involving 91 circulating inflammatory proteins and the risk of PA development.

## Summary

- A causal association between 91 circulating inflammatory proteins and perianal abscess was discovered through a bidirectional Mendelian randomization study involving two samples.
- The research findings suggest that the interleukin‐18 receptor 1 (OR = 1.10, 95% CI 1.01–1.19, *p* = 0.039), interleukin‐33 (OR = 1.31, 95% CI 1.10–1.56, *p* = 0.002), interleukin‐7 (OR = 1.32, 95% CI 1.06–1.64, *p* = 0.014), and tumor necrosis factor ligand superfamily member 12 (OR = 1.16, 95% CI 1.01–1.32, *p* = 0.032) may have negative effects on perianal abscess, while the T‐cell surface glycoprotein CD6 isoform (OR = 0.81, 95% CI 0.68–0.98, *p* = 0.026), interleukin‐2 (OR = 0.80, 95% CI 0.66–0.97, *p* = 0.026), and programmed cell death 1 ligand 1 (OR = 0.84, 95% CI 0.70–1.00, *p* = 0.047) may have protective effects against perianal abscess.
- Reverse Mendelian randomization analysis did not provide evidence of any reverse causal relationships, further supporting the causal association between 91 circulating inflammatory proteins and perianal abscess.

## 1 Introduction

Perianal abscess (PA) is a common benign disease around the anal and rectal area, with an incidence rate of 20 cases per 100,000 people [1]. The incidence rate in males is twice that of females [2]. Due to the fact that some patients may be too embarrassed to seek treatment, or they may self‐treat with antibiotics or wait for the abscess to rupture and subside on its own, the incidence rate is difficult to ascertain accurately, and the actual incidence may be higher [3]. Patients with PA primarily present with perianal pain, and low‐positioned abscesses may be accompanied by perianal swelling and even systemic symptoms such as fever and sepsis [4, 5]. Surgery is currently the best treatment for PA, with the goal of draining the lesion, controlling infection, and preserving the anal sphincter [6]. Some patients may experience anal fistula or recurrent abscess after incision and drainage surgery, which can have a serious impact on their physical, mental, and financial burden [7]. Delving into the pathogenesis and progression mechanisms of PAs and identifying new biomarkers are crucial for accurately assessing the progression and prognosis of the patient's condition.

PA is believed to be caused by infection of the anal glands [2, 4, 8, 9]. In 1956, Eisenhammer proposed the “cryptoglandular theory,” which is now widely accepted [10]. The theory is based on the concept of blockage, where a single gland gets clogged with debris, leading to infection and the development of an abscess [8]. Parks proposed in 1961 that an anal fistula is actually a secondary sinus tract of a diseased anal gland and that 90% of anal fistula are caused by infection of the anal glands [11]. However, in 1967, Goligher and others dissected 60 patients with PA or anal fistulas and found that only 14% or 23% of the patients had intersphincteric abscesses, suggesting that PA or anal fistulas may be related to other mechanisms [12, 13]. Mitalas found that granulation tissue is present in most fistula tissues, suggesting that there is an inflammatory response during the formation of anal fistula [14]. van Onkelen found that bacteria were scarce within fistula tissues and that no mycobacteria were identified. In subsequent studies, it was discovered that significant pro‐inflammatory IL‐1β expression was present in 93% of fistula samples from 27 patients with glandular origin anal fistulas. The expression of IL‐8, IL‐12p40, and TNF‐α was noted in 70%, 33%, and 30% of the fistula specimens, respectively [15, 16]. Ratto and others have found that the cytokines IL‐1β and IL‐8 may play a certain role in the formation of fistulas [17]. Current research on biomarkers shows a strong correlation between inflammation and inflammatory cytokines with the occurrence and development of anal fistulas. However, there are fewer studies on the relationship between inflammation and inflammatory cytokines with the occurrence and progression of PA, and exploring the causal relationship between the two is very necessary for understanding the etiology and treatment of PA.

Mendelian randomization (Mendelianrandomization, MR) was used to study the causal relationship between 91 circulating inflammatory proteins and perianal abscess. MR is a statistical technique used to test the causal relationship between exposure and outcome, which uses genetic variants associated with the exposure as instrumental variables to proxy for the exposure, supporting three assumptions to assess the relationship between the instrumental variable and the outcome [18, 19, 20]. Observational studies commonly used today are susceptible to confounding factors, which refer to all factors other than the study factor that may affect the outcome (including known and unknown factors), and their presence cannot guarantee the accuracy and reliability of study results, nor can they explore the direct causal link between exposure and outcome. Unlike observational studies, MR is not affected by confounding factors because it is based on genetic levels. Therefore, this study adopted a bidirectional two‐sample MR analysis method to explore the bidirectional causal relationship between 91 circulating inflammatory proteins and perianal abscess. Through genetic level analysis, the study aims to reveal the mechanism of the occurrence and progression of perianal abscess, providing a new basis for strengthening prevention and treatment measures.

## 2 Data and Methods

### 2.1 Design

**Figure 1:**
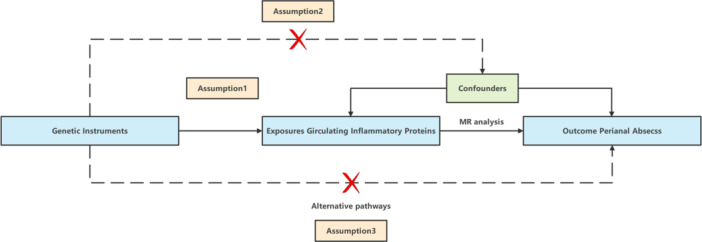
An overview of the MR analysis and its three primary assumptions.

The present study followed the newly established STROBE‐MR statement for reporting MR research [21]. A bidirectional two‐sample MR method was utilized to investigate the potential causal effects of 91 circulating inflammatory proteins on perianal abscess and to conduct a reverse MR study to explore the possibility of a reverse causal relationship. MR is based on three key assumptions [22, 23]: (1) The instrumental variable is closely related to the exposure factor. (2) The instrumental variable should not be influenced by known or unknown confounding factors. (3) The instrumental variable affects the outcome solely through the exposure factor. This study used publicly available data, and therefore, ethical approval and informed consent were not required. The design of this study is illustrated in Figure 1.

### 2.2 Data Resources

The genetic data for 91 circulating inflammatory proteins are derived from 11 cohorts, comprising a total of 14,824 participants. The Olink Inflammation panel was used to measure both the whole‐genome genetic data and the plasma proteomic data. This included a genome‐wide study of quantitative trait loci (pQTLs) for 91 plasma proteins in the 14,824 participants, and the plasma proteins were measured using the Olink Target‐96 Inflammation Immune Analysis panel. The proteomic data from each cohort was generated at the Olink laboratory in Uppsala [24]. The summary statistics for GWAS on perianal abscess is derived from the data set released by FinnGen Consortium R9 [25]. The genetic data related to perianal abscess includes 2595 cases of perianal abscess and 301,931 individuals serving as controls.

### 2.3 Selection of IVs

To select appropriate instrumental variables that comply with the three core Mendelian Randomization (MR) assumptions depicted, a series of quality control methods were employed. Single‐nucleotide polymorphisms (SNPs) closely associated with the exposure factor were chosen with a genome‐wide significance threshold of *p* < 5 × 10^−8^. Unfortunately, only a few SNPs qualified as instrumental factors when selected with a threshold of *p* < 5 × 10^−8^. Consequently, a second threshold was applied by selecting SNPs with a genome‐wide significance threshold of *p* < 1 × 10^−5^ as instrumental variables to explore more potential causal relationships between exposure and outcome [26, 27].

Subsequently, SNPs associated with confounding factors and outcomes with *R*² > 0.001 were filtered out to avoid linkage disequilibrium within a 10,000 kB range. Moreover, the F‐statistic is calculated as [(*n* – *k* − 1)/*k*) × *R*²/(1 – *R*²)], where *R*² represents the variance in exposure explained by the genetic instrument, *k* represents the number of genetic variants, and *n* represents the sample size. *R*² is computed as follows: 2 × β² × EAF × (1 – EAF)/[2 × β² × EAF × (1 – EAF) + se² × 2 × *N* × EAF(1 – EAF)], where EAF denotes the effect allele frequency. Given the relatively lenient thresholds, the F‐statistic was computed for each SNP to confirm its strength and assess the sample overlap effect and weak instrument bias, with an *F* > 10 considered sufficient to mitigate the influence of potential biases [28]. Instrumental variables with an F‐statistic of less than 10 were deemed weak instruments and were excluded from the MR analysis.

Finally, outcome information was extracted from the GWAS catalog database, or FinnGen database, and the relationships between non‐proxy SNPs that satisfied the assumptions were obtained from the results. The exposure data set and outcome data set were merged, which included the relationships between the aforementioned instrumental variables with the outcome and exposure, while palindromic sequences were removed. The remaining SNPs constitute the final instrumental variables for the exposure.

### 2.4 MR Analysis

In this bidirectional two‐sample MR study, five MR analysis methods were employed to validate the bidirectional causal association between 91 circulating inflammatory proteins and perianal abscess, which includes inverse‐variance weighted (IVW) method, weighted median approach, simple mode method, weighted mode method, and MR‐Egger regression, with the inverse‐variance weighted method serving as the gold standard and the results were visually analyzed [29].

### 2.5 Statistical Analysis

The mr_heterogeneity package is used to perform Cochran's Q test on SNPs that meet the null hypothesis to assess heterogeneity between individual genetic variants [30]. If the *p*‐value of Cochran's Q test is less than 0.05, it indicates heterogeneity in the results, suggesting that the relationship between the exposure and the outcome may be influenced by different ages and sexes. In such cases, the final MR results should refer to the inverse‐variance weighted method random effects model as the gold standard. Otherwise, the inverse‐variance weighted method fixed effects model is used as the gold standard. The Mendelian randomization pleiotropy residual sum and outlier (MR‐PRESSO) test and the Egger intercept approach are also used to check if the horizontal MR violates the Mendelian randomization assumptions. For the horizontal pleiotropy Egger‐intercept method, the critical value determines whether genetic variants significantly affect the outcome through pathways other than the exposure, with *p* < 0.05 indicating the presence of horizontal pleiotropy. This signifies that the selected instrumental variables significantly affect the outcome through pathways other than the exposure, violating assumptions 2 and 3. A P‐value greater than 0.05 indicates the exposure does not significantly affect the outcome variable through any pathways other than the exposure itself. As a sensitivity analysis, the leave‐one‐out sensitivity test is employed to infer if any specific SNP in the final set is an outlier. The stability of the results is checked by observing the symmetry in the funnel plot. The MR‐PRESSO method is then used to identify outliers and assess their impact on the results. The bidirectional two‐sample MR analysis is conducted using R software (version 4.3.1).

## 3 Result

### 3.1 Causal Effect of Circulating Inflammatory Proteins on Perianal Abscess

Initially, when exploring the causal relationship between 91 circulating inflammatory proteins and perianal abscess, we adhered to the principles of instrumental variable selection.

**Figure 2:**
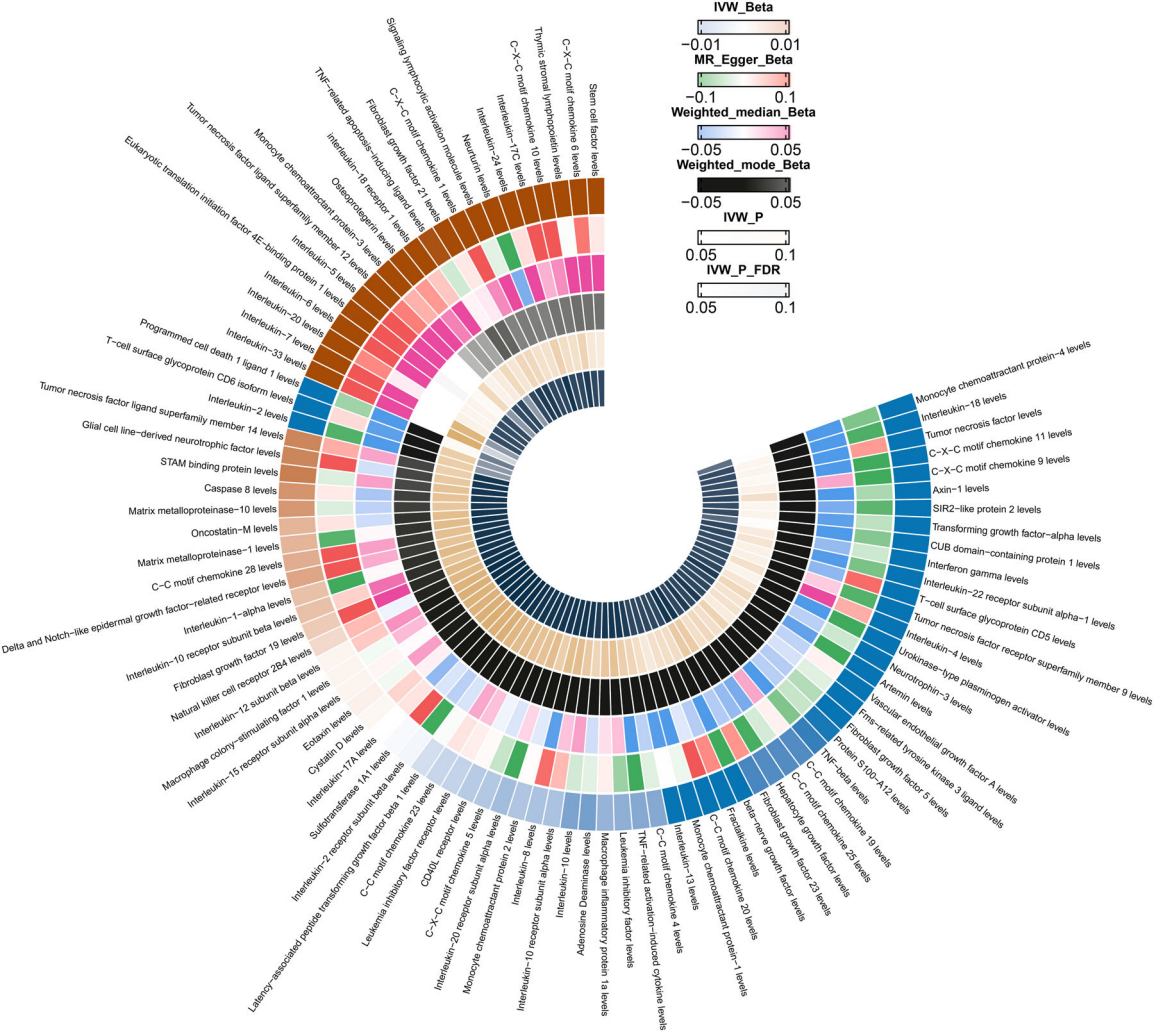
The five outcomes of MR analysis.

**Figure 3:**
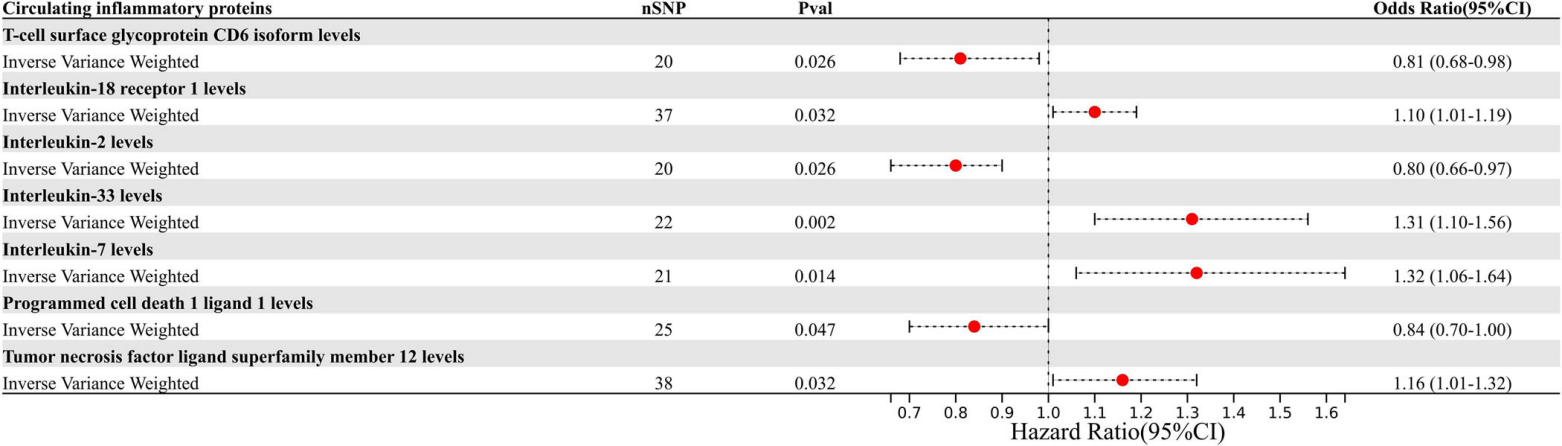
Forest plot summarizing the causal impact of 7 circulating inflammatory proteins on the risk of perianal abscess, based on the IVW method.

**Figure 4:**
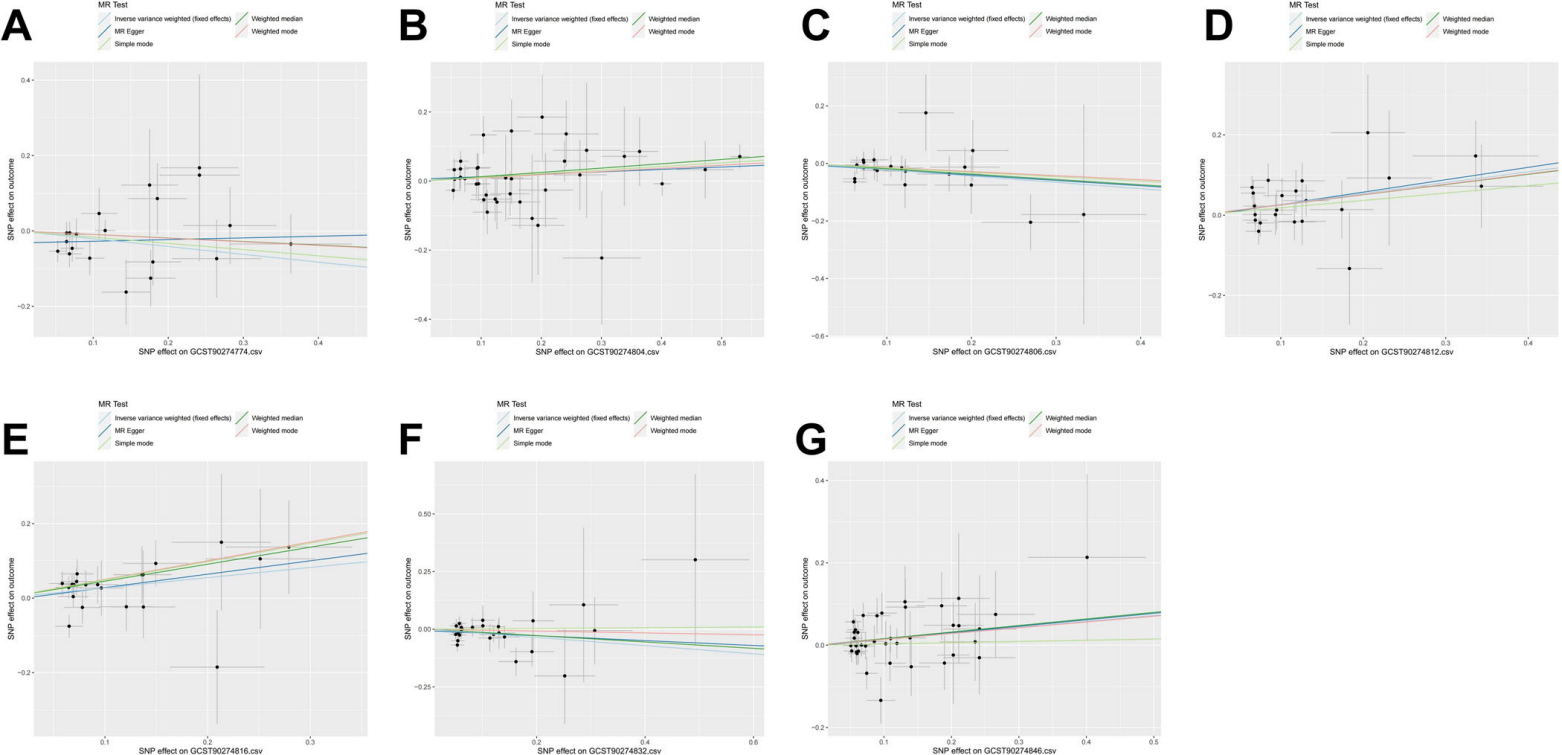
The scatter plots for the association between 91 circulating inflammatory proteins and perianal abscess. (A) T‐cell surface glycoprotein CD6 isoform levels; (B) Interleukin‐18 receptor 1 levels; (C) Interleukin‐2 levels; (D) Interleukin‐33 levels; (E) Interleukin‐7 levels; (F) Programmed cell death 1 ligand 1 levels; (G) Tumor necrosis factor ligand superfamily member 12 levels; Note: SNP effects were plotted into lines for the inverse‐variance weighted test (light blue line), MR‐Egger (blue line), weighted median (green line), Simple mode (light green line) and Weighted mode (red line). The slope of the line corresponded to the causal estimation.

**Figure 5:**
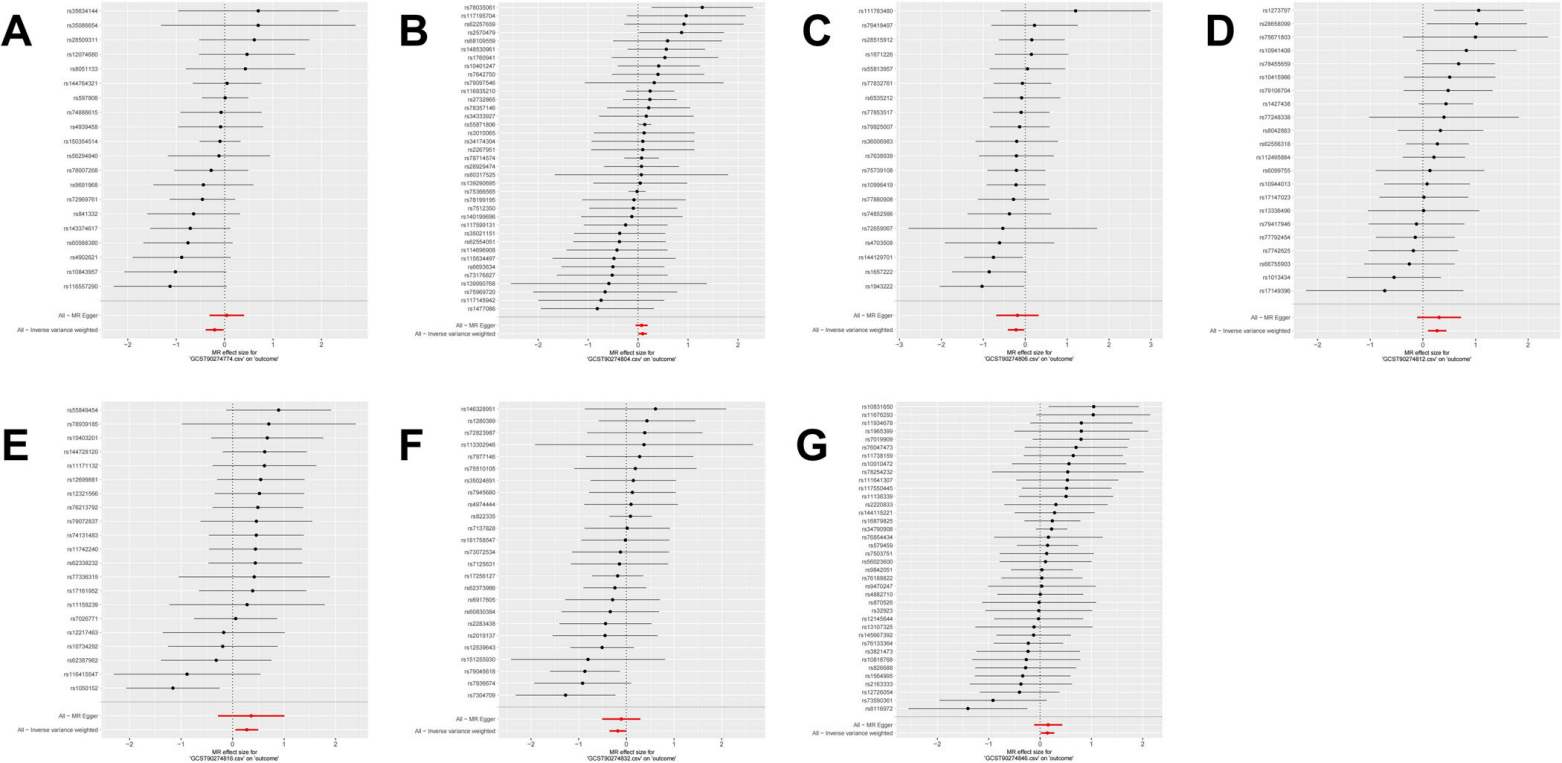
The forest plots for the association between 91 circulating inflammatory proteins and perianal abscess. (A) T‐cell surface glycoprotein CD6 isoform levels; (B) Interleukin‐18 receptor 1 levels; (C) Interleukin‐2 levels; (D) Interleukin‐33 levels; (E) Interleukin‐7 levels; (F) Programmed cell death 1 ligand 1 levels; (G) Tumor necrosis factor ligand superfamily member 12 levels.

We used a two‐sample Mendelian randomization analysis, with the inverse variance weighted (IVW) method as the initial analysis. An overview of all the results can be found in Figure 2. We set a threshold for the false discovery *p*‐value at 0.05. After adjustment for multiple testing based on the *p*‐values, the IVW‐MR results indicate that there are 7 circulating inflammatory proteins that are positively associated with perianal abscess (Figure 3). Among them, 4 factors are circulating inflammatory proteins that are risk factors for perianal abscess, including interleukin‐18 receptor 1 levels (OR = 1.10, CI 1.01–1.19, *p* = 0.039), interleukin‐33 levels (OR = 1.31, CI 1.10–1.56, *p* = 0.002), interleukin‐7 levels (OR = 1.32, CI 1.06–1.64, *p* = 0.014), and tumor necrosis factor ligand superfamily member 12 levels (OR = 1.16, CI 1.01–1.32, *p* = 0.032). In addition, there are 3 circulating inflammatory proteins that serve as protective factors for perianal absecss, including T‐cell surface glycoprotein CD6 isoform levels (OR = 0.81, CI 0.68–0.98, *p* = 0.026), interleukin‐2 levels (OR = 0.80, CI 0.66–0.97, *p* = 0.026), and programmed cell death 1 ligand 1 levels (OR = 0.84, CI 0.70–1.00, *p* = 0.047). We used scatter plots (Figure 4) and forest plots (Figure 5) to visually demonstrate the impact of each circulating inflammatory protein SNP on the risk of perianal abscess. Additionally, the findings of the remaining four computational methods were located in Table 1. Finally, the outcomes of the five MR models, which analyzed the correlation between 91 circulating inflammatory proteins and PA risk, were detailed in Table S1.

**Table 1:** Statistical results of the four models other than IVW.

Circulating inflammatory proteins	Analytical methods	nSNP	*p*‐value	Odds ratio (95% CI)
T‐cell surface glycoprotein CD6 isoform levels				
	MR Egger	20	0.81	1.05 (0.73–1.49)
	Weighted median	20	0.51	0.91 (0.69–1.21)
	Simple mode	20	0.48	0.85 (0.54–1.33)
	Weighted mode	20	0.51	0.91 (0.69–1.20)
Interleukin‐18 receptor 1 levels				
	MR Egger	37	0.26	1.07 (0.95–1.21)
	Weighted median	37	0.03	1.13 (1.01–1.27)
	Simple mode	37	0.47	1.11 (0.84–1.47)
	Weighted mode	37	0.08	1.10 (0.99–1.21)
Interleukin‐2 levels				
	MR Egger	20	0.48	0.83 (0.50–1.38)
	Weighted median	20	0.17	0.83 (0.64–1.08)
	Simple mode	20	0.48	0.86 (0.57–1.30)
	Weighted mode	20	0.47	0.87 (0.59–1.27)
Interleukin‐33 levels				
	MR Egger	22	0.16	1.36 (0.90–2.06)
	Weighted median	22	0.05	1.29 (1.00–1.66)
	Simple mode	22	0.45	1.20 (0.76–1.91)
	Weighted mode	22	0.20	1.29 (0.88–1.90)
Interleukin‐7 levels				
	MR Egger	21	0.29	1.43 (0.75–2.72)
	Weighted median	21	0.002	1.58 (1.17–2.13)
	Simple mode	21	0.08	1.63 (0.98–2.74)
	Weighted mode	21	0.05	1.65 (1.04–2.64)
Programmed cell death 1 ligand 1 levels				
	MR Egger	25	0.61	0.90 (0.60–1.34)
	Weighted median	25	0.28	0.87 (0.68–1.12)
	Simple mode	25	0.94	1.02 (0.65–1.59)
	Weighted mode	25	0.82	0.96 (0.68–1.36)
Tumor necrosis factor ligand superfamily member 12 levels				
	MR Egger	38	0.26	1.17 (0.89–1.54)
	Weighted median	38	0.13	1.17 (0.95–1.44)
	Simple mode	38	0.87	1.03 (0.72–1.48)
	Weighted mode	38	0.28	1.15 (0.90–1.48)

**Figure 6:**
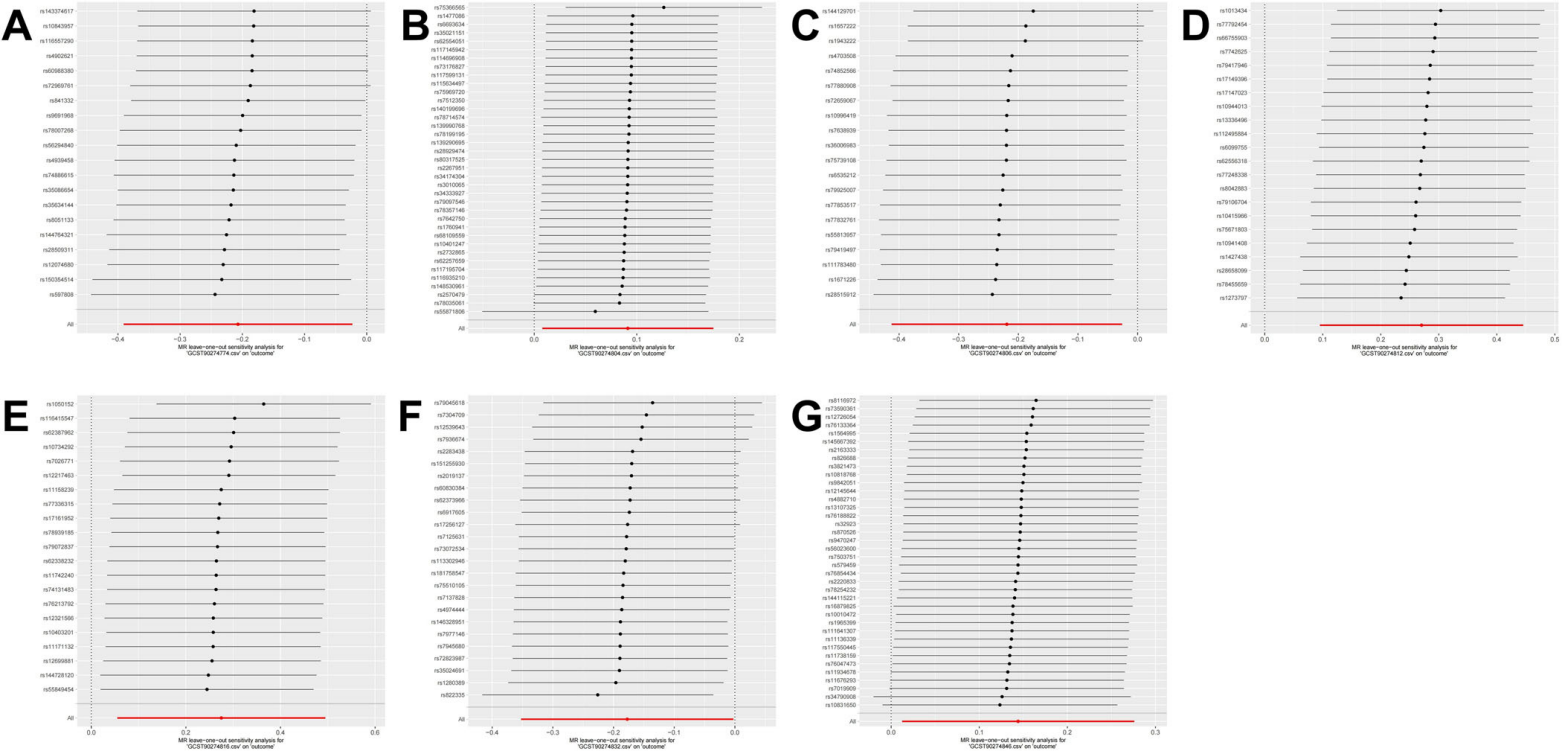
The leave‐one‐out sensitivity analysis for the association between 91 circulating inflammatory proteins and perianal abscess. (A) T‐cell surface glycoprotein CD6 isoform levels; (B) Interleukin‐18 receptor 1 levels; (C) Interleukin‐2 levels; (D) Interleukin‐33 levels; (E) Interleukin‐7 levels; (F) Programmed cell death 1 ligand 1 levels; (G) Tumor necrosis factor ligand superfamily member 12 levels.

**Figure 7:**
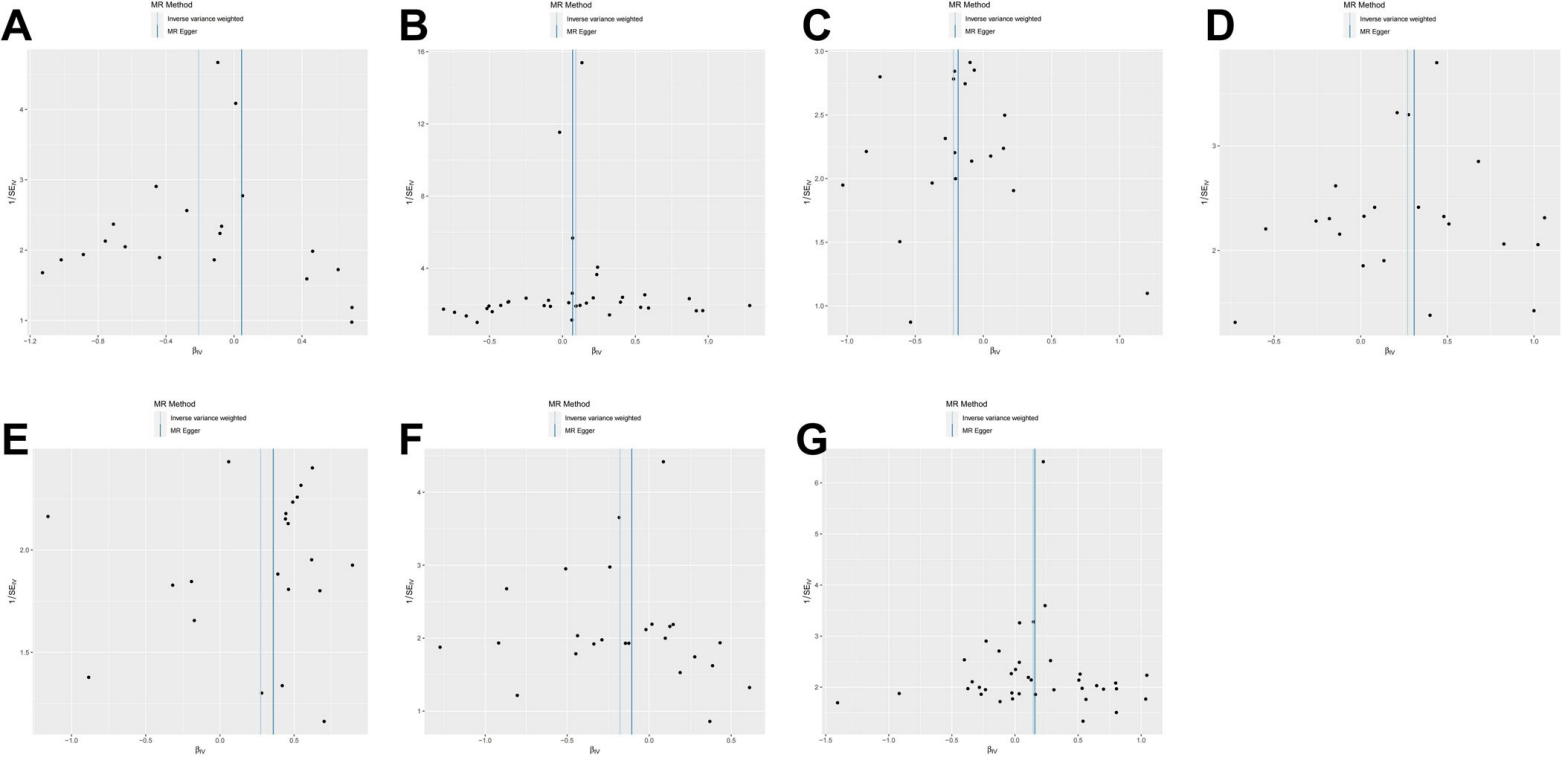
The funnel plots for the association between 91 circulating inflammatory proteins and perianal abscess. (A) T‐cell surface glycoprotein CD6 isoform levels; (B) interleukin‐18 receptor 1 levels; (C) interleukin‐2 levels; (D) interleukin‐33 levels; (E) interleukin‐7 levels; (F) programmed cell death 1 ligand 1 levels; (G) tumor necrosis factor ligand superfamily member 12 levels.

Heterogeneity was evaluated using a Cochran's Q test, employing a distribution of 10,000 for both IVW and MR‐Egger regression methods. Findings showed homogeneity among the SNPs associated with circulating inflammatory proteins for each group (Table 2). The MR‐Egger analysis also did not detect directional pleiotropy within the instrumental variables (*p* > 0.05), confirming the robustness of the causal relationship. Both the leave‐one‐out method (Figure 6) and funnel plots (Figure 7) indicate data reliability.

**Table 2:** Heterogeneity and level of pleiotropy testing for 7 Circulating inflammatory proteins.

Circulating inflammatory proteins	nsnp	Q_inverse. variance. weighted	Q_pval_ inverse. variance. weighted	Egger_intercept	Egger_intercept_pval	MR‐PRESSO_global_test_p
T‐cell surface glycoprotein CD6 isoform levels	20	19.20	0.44	‐0.031	0.12	0.055
Interleukin‐18 receptor 1 levels	37	32.35	0.64	0.006	0.63	0.649
Interleukin‐2 levels	20	12.82	0.85	‐0.003	0.89	0.848
Interleukin‐33 levels	22	20.74	0.47	−0.004	0.84	0.512
Interleukin‐7 levels	21	19.81	0.47	−0.008	0.79	0.478
Programmed cell death 1 ligand 1 levels	25	19.32	0.73	−0.007	0.70	0.745
Tumor necrosis factor ligand superfamily member 12 levels	38	35.92	0.52	−0.002	0.91	0.552

### 3.2 Reverse‐Direction MR Analyses

The IVW approach was utilized to conduct a reverse MR assessment, exploring the potential causal relationship between PA and circulating inflammatory proteins. Our analysis has not found any evidence to suggest a reverse causal relationship between PA and the 7 specific inflammatory factors studied (Table 3). The reliability of our findings was validated through the utilization of the MR‐Egger regression method and the Cochran's Q test.

**Table 3:** Reverse MR analysis results for 7 circulating inflammatory proteins communities.

Circulating inflammatory proteins	OR (95%CI)	Pval	Q_pval_inverse. variance. weighted	Q_pval_MR. egger	Egger_intercept (pval)
Interleukin‐18 receptor 1	0.95 (0.87–1.05)	0.31	0.60	0.78	0.09 (0.51)
Interleukin‐33	0.99 (0.89–1.10)	0.88	0.99	0.89	−0.01 (0.96)
Interleukin‐7	1.00 (0.79–1.28)	> 0.99	0.27	0.46	−0.3 (0.38)
Tumor necrosis factor ligand superfamily member 12	1.00 (0.89–1.11)	0.93	0.88	0.78	−0.05 (0.74)
T‐cell surface glycoprotein CD6 isoform	1.08 (0.98–1.19)	0.14	0.30	0.12	−0.02 (0.92)
Interleukin‐2	0.98 (0.85–1.13)	0.77	0.22	0.52	−0.18 (0.35)
Programmed cell death 1 ligand 1	0.95 (0.77–1.18)	0.66	0.05	0.01	0.02 (0.96)

## 4 Discussion

### 4.1 Main Findings and Interpretation

There is an association between inflammatory proteins and PA, but the causal relationship is not yet clear. It is challenging to determine the causes of changes in inflammatory cytokine levels in patients with perianal abscess through general observational studies. The disease itself, side effects caused by therapeutic drugs, potential pathological immune responses, latent infection status, and harmful lifestyle habits such as alcohol consumption and smoking could all contribute to alterations in inflammatory cytokine levels [31, 32, 33]. A study has found that patients diagnosed with PA have an increased risk of subsequently being diagnosed with Crohn's disease and ulcerative colitis [34]. Litta and others also found that a combination of inflammatory cytokines, chemokines, and growth factors exists in the wound microenvironment of patients with anal fistulas secondary to PA [35]. However, there are currently few studies on PA and inflammatory factors. To our knowledge, this study is the first to comprehensively evaluate the causal effects of 91 inflammatory factors on PA and their reverse impacts. Significant findings were obtained by conducting a bidirectional two‐sample Mendelian Randomization analysis on two independent cohorts. The study identified three inflammatory proteins that may be protective factors for perianal abscess, including T‐cell surface glycoprotein CD6 isoform levels, interleukin‐2 levels, and programmed cell death 1 ligand 1 levels. Additionally, interleukin‐18 receptor 1 levels, interleukin‐33 levels, interleukin‐7 levels, and tumor necrosis factor ligand superfamily member 12 levels may be risk factors in the occurrence and development of PA.

The T cell‐surface glycoprotein CD6 acts as a regulator of cell functions and is associated with the pathogenesis of autoimmune conditions including multiple sclerosis, rheumatoid arthritis, psoriasis, and inflammatory bowel disease [36, 37, 38, 39, 40, 41]. CD6 is a cell surface protein mainly found on T lymphocytes and is involved in the activation and proliferation of immune cells. Catala and colleagues have discovered that triggering monobacterial and polymicrobial sepsis in CD6 (−/−) mice leads to reduced survival probabilities, along with heightened levels of bacterial counts and pro‐inflammatory cytokines [42]. Sarrias et al. present findings that highlight the ectodomain of CD6's capability to bind bacteria, reinforcing its therapeutic promise in treating septic shock syndrome or other infectious inflammatory conditions [43]. Martínez‐Florensa's research indicates that CD6.PD3 initiates a reduction in serum concentrations of pro‐inflammatory cytokines as well as bacteremia. Additionally, CD6.PD3 demonstrates enhanced survival benefits for septic mice when used in conjunction with Imipenem/Cilastatin [44]. Current studies suggest that the levels of T‐cell surface glycoprotein CD6 isoforms may play a protective role in the onset and progression of PA. Interleukin‐18 (IL‐18) is present in areas of persistent inflammation, across a range of autoimmune conditions, within diverse cancer types, and amid numerous infectious disease scenarios [45]. Increased levels of IL‐18 have been documented in inflammatory bowel disease, especially Crohn's disease (CD) [46, 47]. Research suggests that a complete absence of IL‐18 (or its receptor IL‐18R) makes mice more susceptible to intestinal epithelial harm, and creates a modified inflammatory milieu that enhances the potential for intestinal tumor development [48, 49]. IL‐18 can stimulate the release of various other inflammatory cytokines, including IFN‐γ, TNF‐α, IL‐6, and IL‐1β. Interleukin 2 (IL‐2) is a factor produced by T‐cells that plays a crucial role in their activation, growth, and differentiation. It specifically promotes the growth and differentiation of T and B lymphocytes, boosts the function of natural killer (NK) cells, and facilitates the activation of macrophages [50]. IL‐2 plays a protective role in the development of various types of abscesses. High levels of IL‐2 observed after drug treatment can prevent the recurrence of abscesses [51, 52]. Insufficient activity of this critical growth factor may lead to atypical proliferation and clone growth of T‐cells within the intestinal lining, potentially triggering an impaired immune response that results in persistent inflammation observed in conditions such as Crohn's disease and ulcerative colitis [53]. Interleukin‐33 (IL‐33), which belongs to the IL‐1 family, is consistently present and secreted from protective barriers like the cells lining the intestine, acting as a warning signal cytokine [54, 55]. IL‐33 plays a critical role in preserving the integrity of the gut barrier; however, when this barrier is compromised, IL‐33 persists in battling pathogens by drawing in and stimulating innate immune cells, thereby fostering a Type 2 inflammatory response [56]. Some studies indicate that IL‐33 could play a role in immunosuppression during the later stages of sepsis, potentially linked to Treg proliferation and activation, as well as the release of Th2‐type cytokines [57]. Interleukin‐7 (IL‐7) is a unique cytokine mainly produced by epithelial and stromal cells, essential for regulating T cell balance [58]. The study by Belarif et al. suggests a local disruption of IL‐7R signaling in the colons of patients with severe IBD, which may play a role in perpetuating chronic inflammation. Furthermore, elevated IL7 levels in the colon are likely associated with bacterial invasion of tissue [59]. A study demonstrated that IL‐7 rejuvenated the delayed hypersensitivity reaction, reduced lymphocyte apoptosis caused by sepsis, counteracted the suppression of interferon γ (critical for macrophage activation) during sepsis, and enhanced survival in a mouse model of polymicrobial sepsis [60, 61]. Programmed cell death 1 ligand 1 levels (PD‐L1) is found on a range of cells including macrophages, certain T and B cells when activated, mesenchymal stem cells, and also on non‐blood‐forming cells such as liver cells, cells lining blood vessels, epithelial cells, muscle cells, pancreatic beta cells, and brain astrocytes [62, 63]. In mouse studies, PD‐L1 is necessary for preserving the gastrointestinal (GI) tract's integrity and protecting against damage to the epithelial lining during inflammation [64]. Studies with PD‐L1‐deficient mice revealed pronounced ulcers and cellular infiltration, suggesting that the absence of PD‐L1 could promote a more inflammatory condition, resulting in heightened inflammation and damage [64, 65]. However, Teng Zhang and colleagues posited that in sepsis, there is a correlation between PD‐L1 expression and mortality rates, and inhibiting PD‐L1 could provide protection against sepsis. At present, targeting PD‐L1 could potentially reverse the immunosuppression observed in septic conditions [66]. Inhibiting the PD‐1/PD‐L1 pathway to counteract the immune suppression associated with sepsis may offer an encouraging therapeutic strategy [67, 68]. The protein Tumor Necrosis Factor Ligand Superfamily Member 12 (TNFSF12, TWEAK) operates via its receptor, Fn14 (TNFRSF12A or TWEAK‐R), and is involved in various biological functions such as promoting cell proliferation, triggering the release of pro‐inflammatory cytokines, and in some experimental contexts, initiating cell apoptosis [69]. Studies have shown that levels of TWEAK in patients with sepsis are reduced compared to healthy individuals. Additionally, rising TWEAK levels during the progression of the disease are associated with an elevated risk of 28‐day mortality in these patients [70, 71]. Evidence suggests that a deficiency in TWEAK or a decrease in its biological function through the use of anti‐TWEAK monoclonal antibodies can diminish the expression of pro‐inflammatory cytokines and reduce neutrophil and macrophage infiltration. This, in turn, lessens the severity of trinitrobenzenesulfonic (TNBS) acid‐induced colitis in mice, indicating that TWEAK is a potential risk factor in the development and progression of colitis [72].

### 4.2 Limitation

This study, while insightful, has its drawbacks: (1)MR is a powerful tool for causal inference, but it has limitations in practical applications. First, genetic pleiotropy may cause instrumental variables to affect multiple traits simultaneously, introducing bias. Second, MR cannot fully exclude confounding factors, especially those related to exposure or outcome variables. Additionally, the statistical power of MR analysis is influenced by the strength of the instrumental variables, and weak genetic variants may lead to insufficient power. Finally, reverse causality is another challenge in MR analysis. Although reverse MR can be used to detect reverse causality, its results may still be affected by confounding factors. Therefore, these limitations should be carefully considered when using MR to infer causal relationships. (2) Although this study utilized large sample GWAS data, the statistical power to detect causal associations may be lower for certain proteins with weaker genetic instruments, increasing the risk of false negatives. Additionally, there is the issue of false positives due to multiple comparisons. (3) The complexity of human behavior means that understanding genetic susceptibility to a disease provides only a partial strategy for prevention, as the environment also plays a substantial role in disease emergence. MR techniques are limited in their capacity to control for confounding variables, including environmental factors [73]. (4) As the outcome data from the study is drawn from European and American populations, its generalizability needs further confirmation to ensure it reflects a global perspective. (5) Even though the research establishes a causal association between 91 circulating inflammatory proteins and perianal abscess, the specific mechanisms involved are still unclear and warrant further investigation.

## 5 Conclusion

This study found that levels of interleukin‐18 receptor 1 (OR = 1.10, CI 1.01–1.19, *p* = 0.039), interleukin‐33 (OR = 1.31, CI 1.10–1.56, *p* = 0.002), interleukin‐7 (OR = 1.32, CI 1.06–1.64, *p* = 0.014), and tumor necrosis factor ligand superfamily member 12 (OR = 1.16, CI 1.01–1.32, *p* = 0.032) could be potential risk factors in the development of PA. Conversely, T‐cell surface glycoprotein CD6 isoform levels (OR = 0.81, CI 0.68–0.98, *p* = 0.026), interleukin‐2 levels (OR = 0.80, CI 0.66–0.97, *p* = 0.026), and programmed cell death 1 ligand 1 levels (OR = 0.84, CI 0.70‐1.00, *p* = 0.047) may act as protective factors in the occurrence and progression of PA. These findings offer a new perspective on the emergence and development of perianal abscesses and underscore the need for further research to uncover potential pathogenic mechanisms and therapeutic methods.

## Author Contributions

**Zehui Wang:** conceptualization, validation, visualization, writing – original draft. **Tian Chen:** software, supervision. **Hongshuo Shi:** data curation, formal analysis, funding acquisition, investigation, methodology. **Xuecheng Zhang:** conceptualization, methodology, project administration, resources, validation, writing – review and editing. **Wei Yang:** formal analysis, investigation, resources, supervision, visualization, writing – review and editing.

## Conflicts of Interest

The authors declare no conflicts of interest.

## Transparency Statement

The lead authors, Xuecheng Zhang and Wei Yang affirm that this manuscript is an honest, accurate, and transparent account of the study being reported; that no important aspects of the study have been omitted; and that any discrepancies from the study as planned (and, if relevant, registered) have been explained.

## Supporting information

Supplementary Table 1.

## Data Availability

The datasets used and/or analyzed during the current study are available from the corresponding author on reasonable request.
