# Supplementary material for: Unveiling the Intricate Causal Nexus Between 91 Circulating Inflammatory Proteins and Perianal Abscess Through a Comprehensive Bidirectional Two‐Sample Mendelian Randomization Analysis
Source: Health Sci Rep. 2025 May 29;8(6):e70803. doi: 10.1002/hsr2.70803 (PMC12122772; doi:10.1002/hsr2.70803)
Supplement: Supplementary file 1 — Supplementary Table 1. [file HSR2-8-e70803-s001.docx]

| reportedTrait | nsnp | b_MR.Egger | pval_MR.Egger | pval_Weighted.median | pval_Inverse.variance.weighted. | fdr_Inverse.variance.weighted. | pval_Weighted.mode | pval_Simple.mode | F | R2 |
| --- | --- | --- | --- | --- | --- | --- | --- | --- | --- | --- |
| Eukaryotic translation initiation factor 4E-binding protein 1 levels | 18 | 0.233 | 0.387 | 0.383 | 0.173 | 0.845 | 0.592 | 0.857 | 28.746 | 0.037 |
| Adenosine Deaminase levels | 23 | -0.033 | 0.707 | 0.624 | 0.527 | 0.928 | 0.804 | 0.589 | 93.044 | 0.142 |
| Artemin levels | 29 | -0.363 | 0.075 | 0.420 | 0.508 | 0.928 | 0.181 | 0.507 | 21.628 | 0.057 |
| Axin-1 levels | 12 | -0.095 | 0.811 | 0.452 | 0.405 | 0.867 | 0.586 | 0.370 | 22.127 | 0.023 |
| beta-nerve growth factor levels | 33 | -0.482 | 0.028 | 0.171 | 0.438 | 0.867 | 0.433 | 0.896 | 22.976 | 0.055 |
| Caspase 8 levels | 22 | 0.026 | 0.881 | 0.665 | 0.750 | 0.961 | 0.637 | 0.611 | 26.798 | 0.044 |
| Eotaxin levels | 27 | -0.015 | 0.918 | 0.616 | 0.960 | 0.993 | 0.694 | 0.540 | 35.196 | 0.067 |
| C-C motif chemokine 19 levels | 36 | 0.017 | 0.858 | 0.566 | 0.494 | 0.928 | 0.879 | 0.761 | 34.968 | 0.089 |
| C-C motif chemokine 20 levels | 33 | 0.438 | 0.085 | 0.867 | 0.397 | 0.867 | 0.592 | 0.990 | 24.319 | 0.057 |
| C-C motif chemokine 23 levels | 31 | 0.032 | 0.754 | 0.517 | 0.824 | 0.961 | 0.537 | 0.203 | 57.031 | 0.117 |
| C-C motif chemokine 25 levels | 32 | -0.048 | 0.552 | 0.235 | 0.421 | 0.867 | 0.351 | 0.873 | 64.000 | 0.144 |
| C-C motif chemokine 28 levels | 35 | 0.363 | 0.060 | 0.656 | 0.794 | 0.961 | 0.191 | 0.238 | 23.138 | 0.060 |
| C-C motif chemokine 4 levels | 30 | -0.035 | 0.602 | 0.529 | 0.530 | 0.928 | 0.462 | 0.174 | 72.327 | 0.147 |
| Natural killer cell receptor 2B4 levels | 32 | 0.085 | 0.515 | 0.918 | 0.878 | 0.974 | 0.991 | 0.883 | 34.572 | 0.079 |
| CD40L receptor levels | 24 | 0.008 | 0.928 | 0.890 | 0.763 | 0.961 | 0.986 | 0.512 | 64.737 | 0.105 |
| T-cell surface glycoprotein CD5 levels | 27 | 0.178 | 0.377 | 0.566 | 0.207 | 0.845 | 0.923 | 0.091 | 32.464 | 0.064 |
| T-cell surface glycoprotein CD6 isoform levels | 20 | 0.045 | 0.808 | 0.515 | 0.026 | 0.484 | 0.509 | 0.478 | 24.924 | 0.038 |
| CUB domain-containing protein 1 levels | 33 | -0.134 | 0.245 | 0.012 | 0.070 | 0.705 | 0.051 | 0.136 | 42.518 | 0.099 |
| Macrophage colony-stimulating factor 1 levels | 26 | -0.013 | 0.944 | 0.772 | 0.975 | 0.993 | 0.718 | 0.946 | 28.276 | 0.052 |
| Cystatin D levels | 46 | 0.053 | 0.493 | 0.893 | 0.972 | 0.993 | 0.561 | 0.099 | 51.455 | 0.164 |
| Fractalkine levels | 31 | 0.144 | 0.558 | 0.693 | 0.428 | 0.867 | 0.988 | 0.864 | 24.722 | 0.055 |
| C-X-C motif chemokine 1 levels | 22 | 0.211 | 0.198 | 0.504 | 0.490 | 0.928 | 0.621 | 0.318 | 44.489 | 0.068 |
| C-X-C motif chemokine 10 levels | 31 | 0.227 | 0.152 | 0.569 | 0.403 | 0.867 | 0.678 | 0.638 | 31.320 | 0.068 |
| C-X-C motif chemokine 11 levels | 36 | -0.246 | 0.190 | 0.185 | 0.133 | 0.845 | 0.349 | 0.770 | 30.623 | 0.080 |
| C-X-C motif chemokine 5 levels | 20 | -0.064 | 0.516 | 0.769 | 0.760 | 0.961 | 0.653 | 0.842 | 74.928 | 0.101 |
| C-X-C motif chemokine 6 levels | 22 | 0.165 | 0.132 | 0.165 | 0.321 | 0.867 | 0.153 | 0.475 | 73.264 | 0.108 |
| C-X-C motif chemokine 9 levels | 32 | -0.228 | 0.324 | 0.709 | 0.188 | 0.845 | 0.541 | 0.285 | 24.021 | 0.056 |
| Delta and Notch-like epidermal growth factor-related receptor levels | 26 | 0.331 | 0.069 | 0.691 | 0.753 | 0.961 | 0.686 | 0.970 | 37.572 | 0.068 |
| Protein S100-A12 levels | 25 | -0.064 | 0.707 | 0.710 | 0.554 | 0.934 | 0.680 | 0.376 | 28.782 | 0.051 |
| Fibroblast growth factor 19 levels | 31 | 0.277 | 0.160 | 0.358 | 0.853 | 0.970 | 0.102 | 0.493 | 29.356 | 0.064 |
| Fibroblast growth factor 21 levels | 25 | 0.027 | 0.889 | 0.913 | 0.517 | 0.928 | 0.777 | 0.903 | 34.120 | 0.059 |
| Fibroblast growth factor 23 levels | 23 | 0.130 | 0.569 | 0.720 | 0.662 | 0.961 | 0.424 | 0.452 | 25.402 | 0.042 |
| Fibroblast growth factor 5 levels | 34 | -0.085 | 0.297 | 0.429 | 0.274 | 0.867 | 0.384 | 0.893 | 59.615 | 0.166 |
| Fms-related tyrosine kinase 3 ligand levels | 44 | -0.035 | 0.757 | 0.770 | 0.381 | 0.867 | 0.509 | 0.183 | 36.640 | 0.114 |
| Glial cell line-derived neurotrophic factor levels | 20 | 0.234 | 0.122 | 0.800 | 0.688 | 0.961 | 0.891 | 0.434 | 43.398 | 0.059 |
| Hepatocyte growth factor levels | 31 | -0.194 | 0.343 | 0.607 | 0.652 | 0.961 | 0.573 | 0.672 | 25.394 | 0.058 |
| Interferon gamma levels | 19 | -0.056 | 0.756 | 0.605 | 0.276 | 0.867 | 0.918 | 0.952 | 22.335 | 0.041 |
| Interleukin-10 levels | 29 | -0.050 | 0.771 | 0.633 | 0.671 | 0.961 | 0.490 | 0.468 | 27.020 | 0.055 |
| Interleukin-10 receptor subunit alpha levels | 18 | 0.086 | 0.601 | 0.770 | 0.815 | 0.961 | 0.672 | 0.550 | 21.482 | 0.035 |
| Interleukin-10 receptor subunit beta levels | 27 | 0.058 | 0.439 | 0.204 | 0.742 | 0.961 | 0.258 | 0.368 | 71.454 | 0.129 |
| Interleukin-12 subunit beta levels | 34 | 0.067 | 0.407 | 0.304 | 0.938 | 0.993 | 0.388 | 0.705 | 87.214 | 0.212 |
| Interleukin-13 levels | 24 | -0.002 | 0.990 | 0.369 | 0.425 | 0.867 | 0.755 | 0.104 | 21.786 | 0.047 |
| Interleukin-15 receptor subunit alpha levels | 22 | 0.012 | 0.902 | 0.970 | 0.953 | 0.993 | 0.781 | 0.858 | 68.006 | 0.128 |
| Interleukin-17A levels | 18 | 0.034 | 0.880 | 0.630 | 0.999 | 0.999 | 0.487 | 0.605 | 21.769 | 0.035 |
| Interleukin-17C levels | 33 | 0.563 | 0.010 | 0.691 | 0.436 | 0.867 | 0.893 | 0.589 | 22.764 | 0.067 |
| Interleukin-18 levels | 31 | -0.189 | 0.296 | 0.057 | 0.155 | 0.845 | 0.147 | 0.130 | 36.343 | 0.079 |
| interleukin-18 receptor 1 levels | 37 | 0.070 | 0.258 | 0.033 | 0.032 | 0.484 | 0.079 | 0.466 | 82.032 | 0.200 |
| Interleukin-1-alpha levels | 21 | -0.280 | 0.182 | 0.978 | 0.865 | 0.972 | 0.505 | 0.801 | 27.746 | 0.052 |
| Interleukin-2 levels | 20 | -0.185 | 0.481 | 0.166 | 0.026 | 0.484 | 0.475 | 0.475 | 21.808 | 0.038 |
| Interleukin-20 levels | 23 | 0.145 | 0.588 | 0.935 | 0.200 | 0.845 | 0.715 | 0.500 | 21.526 | 0.044 |
| Interleukin-20 receptor subunit alpha levels | 19 | -0.202 | 0.346 | 0.859 | 0.816 | 0.961 | 0.730 | 0.751 | 21.498 | 0.039 |
| Interleukin-22 receptor subunit alpha-1 levels | 20 | -0.146 | 0.538 | 0.565 | 0.351 | 0.867 | 0.165 | 0.178 | 21.343 | 0.039 |
| Interleukin-24 levels | 16 | 0.039 | 0.898 | 0.440 | 0.587 | 0.961 | 0.616 | 0.529 | 21.453 | 0.032 |
| Interleukin-2 receptor subunit beta levels | 21 | -0.251 | 0.283 | 0.764 | 0.975 | 0.993 | 0.678 | 0.979 | 21.251 | 0.040 |
| Interleukin-33 levels | 22 | 0.308 | 0.161 | 0.051 | 0.002 | 0.226 | 0.204 | 0.445 | 21.124 | 0.043 |
| Interleukin-4 levels | 20 | 0.102 | 0.699 | 0.389 | 0.393 | 0.867 | 0.399 | 0.475 | 21.368 | 0.038 |
| Interleukin-5 levels | 23 | 0.306 | 0.101 | 0.277 | 0.115 | 0.845 | 0.490 | 0.726 | 21.433 | 0.044 |
| Interleukin-6 levels | 13 | 0.262 | 0.254 | 0.373 | 0.167 | 0.845 | 0.500 | 0.539 | 35.532 | 0.033 |
| Interleukin-7 levels | 21 | 0.359 | 0.287 | 0.003 | 0.014 | 0.484 | 0.048 | 0.077 | 21.737 | 0.033 |
| Interleukin-8 levels | 28 | 0.177 | 0.316 | 0.476 | 0.806 | 0.961 | 0.284 | 0.367 | 23.837 | 0.048 |
| Latency-associated peptide transforming growth factor beta 1 levels | 30 | -0.007 | 0.961 | 0.833 | 0.888 | 0.974 | 0.758 | 0.769 | 28.509 | 0.060 |
| Leukemia inhibitory factor levels | 25 | -0.110 | 0.610 | 0.775 | 0.764 | 0.961 | 0.581 | 0.493 | 21.065 | 0.048 |
| Leukemia inhibitory factor receptor levels | 26 | 0.021 | 0.881 | 0.724 | 0.824 | 0.961 | 0.896 | 0.394 | 35.502 | 0.080 |
| Monocyte chemoattractant protein-1 levels | 28 | -0.020 | 0.874 | 0.277 | 0.295 | 0.867 | 0.481 | 0.638 | 38.590 | 0.075 |
| Monocyte chemoattractant protein 2 levels | 45 | 0.004 | 0.943 | 0.715 | 0.610 | 0.961 | 0.449 | 0.876 | 84.915 | 0.257 |
| Monocyte chemoattractant protein-3 levels | 24 | 0.096 | 0.601 | 0.162 | 0.193 | 0.845 | 0.276 | 0.451 | 30.335 | 0.064 |
| Monocyte chemoattractant protein-4 levels | 26 | -0.142 | 0.270 | 0.239 | 0.059 | 0.669 | 0.416 | 0.815 | 52.809 | 0.094 |
| Macrophage inflammatory protein 1a levels | 18 | 0.020 | 0.859 | 0.813 | 0.704 | 0.961 | 0.785 | 0.308 | 78.708 | 0.097 |
| Matrix metalloproteinase-1 levels | 25 | -0.180 | 0.340 | 0.974 | 0.843 | 0.970 | 0.360 | 0.350 | 32.843 | 0.058 |
| Matrix metalloproteinase-10 levels | 23 | -0.039 | 0.751 | 0.685 | 0.709 | 0.961 | 0.769 | 0.850 | 60.132 | 0.096 |
| Neurturin levels | 24 | -0.263 | 0.079 | 0.491 | 0.390 | 0.867 | 0.392 | 0.592 | 21.325 | 0.050 |
| Neurotrophin-3 levels | 31 | -0.056 | 0.784 | 0.699 | 0.337 | 0.867 | 0.998 | 0.964 | 22.280 | 0.050 |
| Osteoprotegerin levels | 27 | 0.126 | 0.461 | 0.511 | 0.269 | 0.867 | 0.711 | 0.848 | 35.945 | 0.067 |
| Oncostatin-M levels | 22 | 0.032 | 0.868 | 0.800 | 0.820 | 0.961 | 0.780 | 0.846 | 27.413 | 0.043 |
| Programmed cell death 1 ligand 1 levels | 25 | -0.106 | 0.606 | 0.280 | 0.047 | 0.607 | 0.823 | 0.944 | 26.094 | 0.049 |
| Stem cell factor levels | 45 | 0.027 | 0.784 | 0.033 | 0.272 | 0.867 | 0.053 | 0.518 | 36.895 | 0.116 |
| SIR2-like protein 2 levels | 20 | -0.176 | 0.366 | 0.540 | 0.164 | 0.845 | 0.638 | 0.127 | 26.902 | 0.038 |
| Signaling lymphocytic activation molecule levels | 33 | -0.031 | 0.863 | 0.363 | 0.394 | 0.867 | 0.389 | 0.741 | 25.825 | 0.062 |
| Sulfotransferase 1A1 levels | 31 | 0.396 | 0.028 | 0.802 | 0.982 | 0.993 | 0.655 | 0.842 | 26.562 | 0.074 |
| STAM binding protein levels | 18 | -0.038 | 0.907 | 0.746 | 0.758 | 0.961 | 0.874 | 0.552 | 21.867 | 0.028 |
| Transforming growth factor-alpha levels | 24 | -0.074 | 0.729 | 0.480 | 0.182 | 0.845 | 0.691 | 0.742 | 24.085 | 0.042 |
| Tumor necrosis factor levels | 27 | 0.124 | 0.531 | 0.232 | 0.170 | 0.845 | 0.994 | 0.954 | 22.237 | 0.056 |
| TNF-beta levels | 41 | -0.140 | 0.107 | 0.044 | 0.405 | 0.867 | 0.081 | 0.330 | 51.574 | 0.179 |
| Tumor necrosis factor receptor superfamily member 9 levels | 34 | -0.183 | 0.230 | 0.754 | 0.214 | 0.845 | 0.669 | 0.707 | 25.303 | 0.078 |
| Tumor necrosis factor ligand superfamily member 14 levels | 34 | 0.102 | 0.418 | 0.618 | 0.610 | 0.961 | 0.431 | 0.153 | 32.315 | 0.097 |
| TNF-related apoptosis-inducing ligand levels | 35 | -0.052 | 0.522 | 0.923 | 0.337 | 0.867 | 0.991 | 0.277 | 55.649 | 0.136 |
| TNF-related activation-induced cytokine levels | 42 | -0.285 | 0.023 | 0.173 | 0.614 | 0.961 | 0.065 | 0.374 | 36.486 | 0.107 |
| Thymic stromal lymphopoietin levels | 22 | -0.005 | 0.983 | 0.278 | 0.544 | 0.934 | 0.385 | 0.771 | 22.076 | 0.044 |
| Tumor necrosis factor ligand superfamily member 12 levels | 38 | 0.158 | 0.263 | 0.133 | 0.032 | 0.484 | 0.279 | 0.872 | 32.220 | 0.086 |
| Urokinase-type plasminogen activator levels | 38 | -0.229 | 0.104 | 0.118 | 0.182 | 0.845 | 0.224 | 0.277 | 29.593 | 0.080 |
| Vascular endothelial growth factor A levels | 29 | 0.016 | 0.872 | 0.573 | 0.364 | 0.867 | 0.437 | 0.421 | 57.581 | 0.113 |
|  |  |  |  |  |  |  |  |  |  |  |
|  |  |  |  |  |  |  |  |  |  |  |
|  |  |  |  |  |  |  |  |  |  |  |
|  |  |  |  |  |  |  |  |  |  |  |
|  |  |  |  |  |  |  |  |  |  |  |
|  |  |  |  |  |  |  |  |  |  |  |
|  |  |  |  |  |  |  |  |  |  |  |
|  |  |  |  |  |  |  |  |  |  |  |
|  |  |  |  |  |  |  |  |  |  |  |
|  |  |  |  |  |  |  |  |  |  |  |
|  |  |  |  |  |  |  |  |  |  |  |
|  |  |  |  |  |  |  |  |  |  |  |
|  |  |  |  |  |  |  |  |  |  |  |
|  |  |  |  |  |  |  |  |  |  |  |
|  |  |  |  |  |  |  |  |  |  |  |
|  |  |  |  |  |  |  |  |  |  |  |
|  |  |  |  |  |  |  |  |  |  |  |
|  |  |  |  |  |  |  |  |  |  |  |
|  |  |  |  |  |  |  |  |  |  |  |
|  |  |  |  |  |  |  |  |  |  |  |
|  |  |  |  |  |  |  |  |  |  |  |
|  |  |  |  |  |  |  |  |  |  |  |
|  |  |  |  |  |  |  |  |  |  |  |
|  |  |  |  |  |  |  |  |  |  |  |
|  |  |  |  |  |  |  |  |  |  |  |
|  |  |  |  |  |  |  |  |  |  |  |
|  |  |  |  |  |  |  |  |  |  |  |
|  |  |  |  |  |  |  |  |  |  |  |
|  |  |  |  |  |  |  |  |  |  |  |
|  |  |  |  |  |  |  |  |  |  |  |
|  |  |  |  |  |  |  |  |  |  |  |
|  |  |  |  |  |  |  |  |  |  |  |
|  |  |  |  |  |  |  |  |  |  |  |
|  |  |  |  |  |  |  |  |  |  |  |
|  |  |  |  |  |  |  |  |  |  |  |
|  |  |  |  |  |  |  |  |  |  |  |
|  |  |  |  |  |  |  |  |  |  |  |
|  |  |  |  |  |  |  |  |  |  |  |
|  |  |  |  |  |  |  |  |  |  |  |
|  |  |  |  |  |  |  |  |  |  |  |
|  |  |  |  |  |  |  |  |  |  |  |
|  |  |  |  |  |  |  |  |  |  |  |
|  |  |  |  |  |  |  |  |  |  |  |
|  |  |  |  |  |  |  |  |  |  |  |
|  |  |  |  |  |  |  |  |  |  |  |
|  |  |  |  |  |  |  |  |  |  |  |
|  |  |  |  |  |  |  |  |  |  |  |
|  |  |  |  |  |  |  |  |  |  |  |
|  |  |  |  |  |  |  |  |  |  |  |
|  |  |  |  |  |  |  |  |  |  |  |
|  |  |  |  |  |  |  |  |  |  |  |
|  |  |  |  |  |  |  |  |  |  |  |
|  |  |  |  |  |  |  |  |  |  |  |
|  |  |  |  |  |  |  |  |  |  |  |
|  |  |  |  |  |  |  |  |  |  |  |
|  |  |  |  |  |  |  |  |  |  |  |
|  |  |  |  |  |  |  |  |  |  |  |
|  |  |  |  |  |  |  |  |  |  |  |
|  |  |  |  |  |  |  |  |  |  |  |
|  |  |  |  |  |  |  |  |  |  |  |
|  |  |  |  |  |  |  |  |  |  |  |
|  |  |  |  |  |  |  |  |  |  |  |
|  |  |  |  |  |  |  |  |  |  |  |
|  |  |  |  |  |  |  |  |  |  |  |
|  |  |  |  |  |  |  |  |  |  |  |
|  |  |  |  |  |  |  |  |  |  |  |
|  |  |  |  |  |  |  |  |  |  |  |
|  |  |  |  |  |  |  |  |  |  |  |
|  |  |  |  |  |  |  |  |  |  |  |
|  |  |  |  |  |  |  |  |  |  |  |
|  |  |  |  |  |  |  |  |  |  |  |
|  |  |  |  |  |  |  |  |  |  |  |
|  |  |  |  |  |  |  |  |  |  |  |
|  |  |  |  |  |  |  |  |  |  |  |
|  |  |  |  |  |  |  |  |  |  |  |
|  |  |  |  |  |  |  |  |  |  |  |
|  |  |  |  |  |  |  |  |  |  |  |
|  |  |  |  |  |  |  |  |  |  |  |
|  |  |  |  |  |  |  |  |  |  |  |
|  |  |  |  |  |  |  |  |  |  |  |
|  |  |  |  |  |  |  |  |  |  |  |
